# Supplementary figures and images for: Uncovering candidate genes responsive to salt stress in Salix matsudana (Koidz) by transcriptomic analysis
Source: PLoS One. 2020 Aug 6;15(8):e0236129. doi: 10.1371/journal.pone.0236129 (PMC7410171; doi:10.1371/journal.pone.0236129)

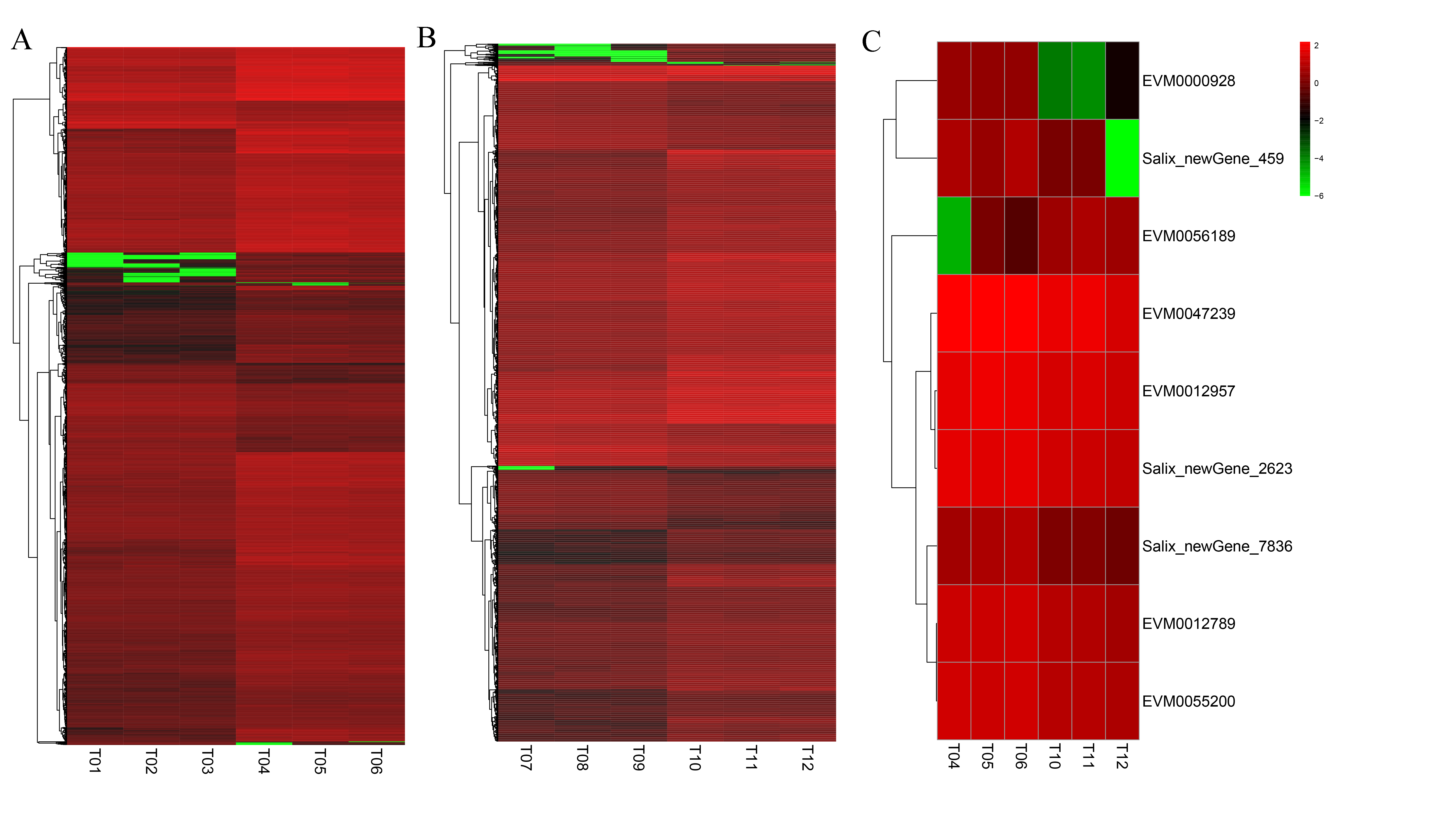

Supplement: S1 Fig — (TIF) [file pone.0236129.s001.tif]

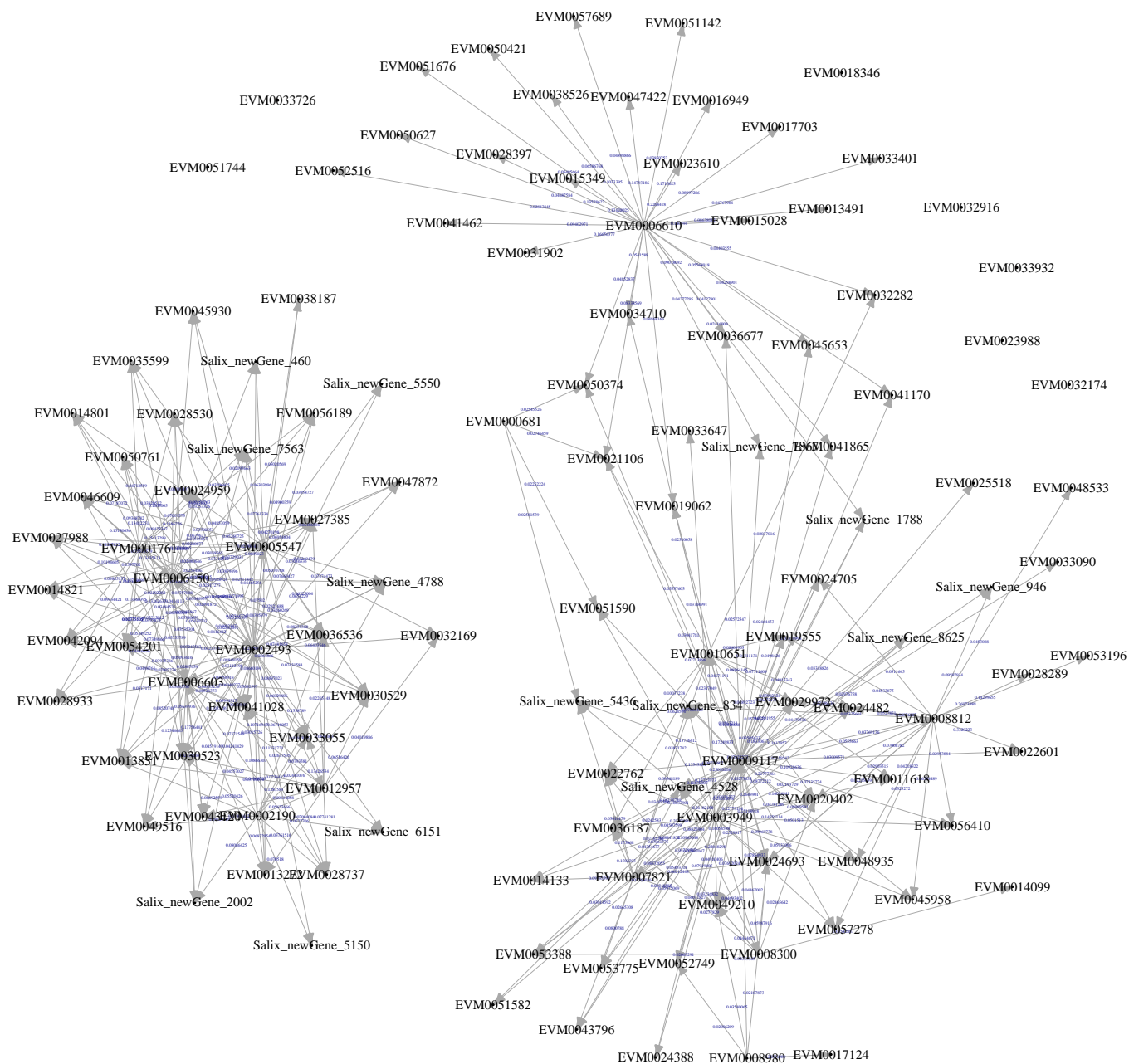

Supplement: S2 Fig — (PDF) [file pone.0236129.s002.pdf]
