# Supplementary material for: Uncovering candidate genes responsive to salt stress in Salix matsudana (Koidz) by transcriptomic analysis
Source: PLoS One. 2020 Aug 6;15(8):e0236129. doi: 10.1371/journal.pone.0236129 (PMC7410171; doi:10.1371/journal.pone.0236129)
Supplement: S1 Table — (DOCX) [file pone.0236129.s003.docx]

S1Table qRT-PCR Primer list

| Gene name | Primer name | Primer Sequence |
| --- | --- | --- |
| EVM0028859 | QWRKY-U1-F | ACAGCTATCAGCAACAGCAG |
|  | QWRKY-U1-R | TCCGTATGCAGAGGGAAAAGAG |
| EVM0057622 | QWRKY-U2-F | AACCAACCGGACATGTTTCC |
|  | QWRKY-U2-R | TGACCGGAGTTGCGATTTTC |
| EVM0032514 | QMYB-U1-F | AGTTGCTGCAAGTGAATCCG |
|  | QMYB-U1-R | AAACCTGTGCATCCAAAGCG |
| EVM0028590 | QMYB-U2-F | TTGTCAGACGCTTTGGATGC |
|  | QMYB-U2-R | CCAGAATCATTTTCCGGGCATG |
| EVM0007050 | QMYB-D1-F | TGAGCCTGCTCCTGTTCTTG |
|  | QMYB-D1-R | AACTGGCTGTGTCAGAAACC |
| EVM0003580 | QbZIP1-U1-F | TGGAGGATCTGGTCAATGAAGC |
|  | QbZIP1-U1-R | AGCCTCTCGGTCAATTCGAC |
| EVM0049031 | QbZIP1-D1-F | GCTCCTCAGCTTGAACTCAAAG |
|  | QbZIP1-D1-R | TCAATTGAGACGGTCTCTCTGC |
| EVM0011014 | QNAC-U1-F | TGGTGATGAGTGATGCCAGTG |
|  | QNAC-U1-R | ACCTCCTTTTCGCATGTGAC |
| EVM0047239 | 9DEG-U1-F | ACAAGCCAACATGGTTGCAC |
|  | 9DEG-U1-R | ACTCTTCCACCATTGGTTGC |
| SapurV1A.0655s0050.1 | Sp Actin1-Q-F | GTCAAGTTCTTTGCTTTCCTCC |
|  | Sp Actin1-Q-F | CATCACAATCACTCTCCGACTA |
