# Supplementary material for: Uncovering candidate genes responsive to salt stress in Salix matsudana (Koidz) by transcriptomic analysis
Source: PLoS One. 2020 Aug 6;15(8):e0236129. doi: 10.1371/journal.pone.0236129 (PMC7410171; doi:10.1371/journal.pone.0236129)
Supplement: S3 Table — (DOCX) [file pone.0236129.s005.docx]

S3 Table The annotation of GO, KEGG pathway and NR of DEGs in salt-sensitive and salt-tolerant groups under 150mM salt stress treatment compared with the whole genome of *S. matsudana*

| GeneID | GO_ | KEGG_pathway_annotation | NR_annotation |
| --- | --- | --- | --- |
| EVM0000928  (S-0928） | -- | Protein export (ko03060);; Protein processing in endoplasmic reticulum (ko04141) | Translocation protein sec62, putative [Ricinus communis] |
| EVM0012789  (S-2789) | Cellular Component: vacuole (GO:0005773);; Cellular Component: peroxisome (GO:0005777);; Molecular Function: FMN binding (GO:0010181);; Cellular Component: membrane (GO:0016020);; Cellular Component: apoplast (GO:0048046);; Biological Process: oxidation-reduction process (GO:0055114);; | Glyoxylate and dicarboxylate metabolism (ko00630);; Peroxisome (ko04146) | PREDICTED: peroxisomal (S)-2-hydroxy-acid oxidase-like [Populus euphratica] |
| EVM0012957  (S-2957) | Biological Process: response to sucrose (GO:0009744);; Biological Process: negative regulation of reductive pentose-phosphate cycle (GO:0080153);; | -- | PREDICTED: calvin cycle protein CP12-1, chloroplastic [Populus euphratica] |
| EVM0047239  (S-7239) | Molecular Function: ribulose-bisphosphate carboxylase activity (GO:0016984);;Cellular Component: cytosolic ribosome (GO:0022626);;Cellular Component: apoplast (GO:0048046);; Biological Process: oxidation-reduction process (GO:0055114);; | Glyoxylate and dicarboxylate metabolism (ko00630);; Carbon fixation in photosynthetic organisms (ko00710);; Carbon metabolism (ko01200) | Ribulose bisphosphate carboxylase small chain 1A family protein [Populus trichocarpa] |
| EVM0055200  (S-5200) | -- | Carbon fixation in photosynthetic organisms (ko00710);; Carbon metabolism (ko01200) | Sedoheptulose-1 family protein [Populus trichocarpa] |
| EVM0056189  (S-6189) | Molecular Function: structural constituent of ribosome (GO:0003735);; Cellular Component: ribosome (GO:0005840);; Biological Process: translation (GO:0006412);; | Ribosome (ko03010) | PREDICTED: 60S ribosomal protein L12-like [Populus euphratica] |
| Salix_newGene_2623  (S-n2623) | Biological Process: reductive pentose-phosphate cycle (GO:0019253);; Cellular Component: cytosolic ribosome (GO:0022626);; Cellular Component: apoplast (GO:0048046);; Biological Process: oxidation-reduction process (GO:0055114);; | Glyoxylate and dicarboxylate metabolism (ko00630);; Carbon fixation in photosynthetic organisms (ko00710);; Carbon metabolism (ko01200) | PREDICTED: ribulose bisphosphate carboxylase small chain, chloroplastic-like [Populus euphratica] |
| Salix_newGene_0459  (S-n459) | Molecular Function: DNA binding (GO:0003677);; Molecular Function: sequence-specific DNA binding transcription factor activity (GO:0003700);; Biological Process: regulation of transcription, DNA-templated (GO:0006355);; | -- | PREDICTED: AP2-like ethylene-responsive transcription factor ANT isoform X2 [Populus euphratica] |
| Salix_newGene_7836  (S-n7836) | Biological Process: response to cold (GO:0009409);; (GO:0009570);; Cellular Component: integral component of membrane (GO:0016021);; Biological Process: hydrogen peroxide catabolic process (GO:0042744);; Cellular Component: apoplast (GO:0048046);; | Carbon fixation in photosynthetic organisms (ko00710);; Carbon metabolism (ko01200) | hypothetical protein POPTR_0005s27550g [Populus trichocarpa] |
