# Supplementary material for: Uncovering candidate genes responsive to salt stress in Salix matsudana (Koidz) by transcriptomic analysis
Source: PLoS One. 2020 Aug 6;15(8):e0236129. doi: 10.1371/journal.pone.0236129 (PMC7410171; doi:10.1371/journal.pone.0236129)
Supplement: Attachment — Submitted filename: Response to Reviewers.docx [file pone.0236129.s009.docx]

Response to Reviewers

Response to editor

1. We edited the manuscript to meet PLOS ONE's style requirements, including file name.
2. We rephrased the overlapping text (three small sections were found) in the manuscript and quoted the necessary reference.
3. We copyedited the language usage, spelling, and grammar in my manuscript using a professional scientific editing service from Editage Company.

According to the editor, the details of the professional service were provided and the copy of manuscript with track changes was uploaded as a *supporting information* file).

4. The author Guoyuan Liu was included in the manuscript submission data.
5. There is no author named Guoliang Liu in our manuscript, which is a mistake in the first time submission.

Response to Reviewer's 1

1. We provided specific manufacturers and country to the Nikon Z50 instrument according to review’s suggestion.
2. The conference genome we mentioned in the manuscript is genome *Salix matsudana* which sequenced recently in our lab but not published yet.
3. The SBPase is an enzyme coded by a DEG selected from pairwise combination salt-treated salt-sensitive vs salt-treated salt-tolerant. It was previously reported that this gene was important players in mediating salt tolerance in Arabidopsis, so we selected this gene to do further functional analysis. In many previous reports, researchers usually use Arabidopsis or tobacco to do ectopic functional verification on some genes from species which can’t obtain transgenic plants. There are no proper yeast strains to test the gene’s function in our lab, so we directly using the Arabidopsis. In the resubmission manuscript, we add the data concerning the phenotype of transgenic Arabidopsis plant, such as the survival rate and chlorophyll content under salt stress.
4. As what the reviewer’s said, the role of SBPase gene is concerning the carbohydrate metabolism and osmotic –regulation. The Fig 8A demonstrated the important players in salt stress signaling pathway and corresponded the result part “Identification of DEGs coding for important components of the salt stress response network”. So we didn’t include the SBPase gene in the Fig 8A.
5. According to the reviewer’s comments, using transcriptome data, the Weighted gene co-expression network (WGCNA) analysis was performed and several hub genes were revealed in the new submission （S2 Figure）, which will guide our future work in gene functional analysis.

Response to Reviewer's 2

1. We edited the language usage, spelling, and grammar in my manuscript and corrected the inconsistencies and errors reviewer mentioned. “Yanjiang” is the correct name for the salt-sensitive *Salix matsudana* material.
2. We realized that using “9901” and “Yanjiang” in the result is not reasonable for the salt-tolerant and salt-sensitive material, we have edited the description in the resubmission manuscript, Salt sensitive (SS) refers to Yanjiang and salt-sensitive lines from F1; Salt tolerant (SN) refers to 9901 and salt-tolerant lines from F1.
3. In the figure 1, the stems of salt-tolerant and salt-sensitive lines were directly immersed into the 100mMol NaCl, it’s difficult for the stems to root in this concentration. But 9901 can shoot normally, which showed the difference capability in salt tolerance. Without salt stress, all stems can root normally. In the RNA sequencing, the experiments were carried out as the description in Materials and methods, after culture in solution without NaCl for 20d, the roots were generated and the stems with roots were transferred to 150 mM NaCl to do salt stress treatment.
4. The root samples were used in qRT-PCR. The root samples came from the repeated experiments which mimic the RNA sequencing material collection procedure.
5. According to the reviewer’s comments, we added the network diagram created by Cytoscape in the new submission and the reference was also added in the reference list.
6. According to the reviewer’s comments, more analysis on the survival rate, electrolyte leakage, SBPase activity and chlorophyll content under salt stress of transgenic Arabidopsis plant were made. In our experiment, the 3 weeks seedlings of WT and overexpression lines were watering with 200mM NaCl for 2 Weeks, but no evident difference in survival rate was found, almost all seedlings are alive (Perhaps because of the short treatment time or low NaCl concentration). Relative electrolyte leakage was increased both in WT and overexpression lines, and no difference was not found either. The only difference was found in chlorophyll content; the content in overexpression lines was higher than that in WT lines after salt treatment.
7. Only depending on the expression pattern, it’s hard to make deep prediction of functions of TFs discovered in the RNA-seq, but we rewrite this part in the new submission to make some comparison and detailed discussion. Uncovering the important roles of TFs under salt and drought stress within willow species is our aims of future research.
8. We agree with the opinion of reviewer that “other DEGs in “9901” CK vs “9901” NT may also account for the stronger salt tolerance”. In the manuscript, we made some discussion on this part DEGs including five different TF family members and player’s in salt stress signaling pathway.
