# Supplementary material for: Uncovering candidate genes responsive to salt stress in Salix matsudana (Koidz) by transcriptomic analysis
Source: PLoS One. 2020 Aug 6;15(8):e0236129. doi: 10.1371/journal.pone.0236129 (PMC7410171; doi:10.1371/journal.pone.0236129)
Supplement: Attachment — Submitted filename: Response to 2nd Reviewers.docx [file pone.0236129.s010.docx]

Response to 2nd Reviewers’ comments

Response to editor

1.	Photos of ‘9901’ and ‘Yanjiang’ materials which cultured under normal growth condition were placed in Fig1 as Fig1 E and Fig1 F respectively. The shoots and roots of both materials regenerated normally after 15d culture in Hoagland medium.

2.	We provided pictures of the two transgenic Arabidopsis lines in comparison to the WT to show salt tolerance under salt stress condition in the new submission. In the experiment, the seeds of WT and two transgenic Arabidopsis lines were planted on MS medium supplied with 0mM NaCl, 50mM NaCl, 75mM NaCl, 100mM NaCl respectively and cultured for 7 days. As control, there was no difference in the seed germination time and seedling growth phenotype between WT and two transgenic Arabidopsis lines when grown under normal condition (0mM NaCl), but when the seeds were cultured under salt stress condition, the two transgenic Arabidopsis lines showed high salt tolerance capability with earlier germination and higher growth rate in medium with higher salt content. These pictures were placed in Fig9 as Fig9 H.

3.	We rechecked the English writing and edited the mistakes that mentioned by editor and we also check other mistakes in the manuscript including legends and method section.

According to the editor, the copy of manuscript with track changes was uploaded as a *supporting information* file).

Response to Reviewer's 2

1. In the new submission, the images of ‘9901’ and ‘Yanjiang’ materials which cultured under normal growth condition were used as control and placed in Fig1 as Fig1 E and Fig1 F respectively.
2. In the new submission, we changed the experiment strategy by planting the seeds directly on MS medium supplied with 0mM NaCl, 50mM NaCl, 75mM NaCl, 100mM NaCl respectively and cultured for 7 days. The phenotype differences were recorded and the pictures were placed in Fig9 as Fig9 H. Results showed that compared with WT plants, the two transgenic Arabidopsis lines had high salt tolerance capability with earlier germination and higher growth rate in medium with higher salt content.
3. In the manuscript, Arabidopsis ecotype Col-0 was used to do transformation experiments.
